# Mediterranean Diet, Sustainability, and Tourism—A Study of the Market’s Demand and Knowledge

**DOI:** 10.3390/foods12132463

**Published:** 2023-06-23

**Authors:** Marzia Ingrassia, Luca Altamore, Pietro Columba, Sara Raffermati, Giuseppe Lo Grasso, Simona Bacarella, Stefania Chironi

**Affiliations:** Department of Agricultural, Food and Forestry Sciences, Università degli Studi di Palermo, 90123 Palermo, Italy; luca.altamore@unipa.it (L.A.); pietro.columba@unipa.it (P.C.); sara.raffermati@community.unipa.it (S.R.); giuseppe.lograsso03@community.unipa.it (G.L.G.); simona.bacarella@unipa.it (S.B.); stefania.chironi@unipa.it (S.C.)

**Keywords:** Mediterranean diet pattern, UNESCO heritage, sustainable food systems, quality food products, food policy, food security, communication model, agribusiness system marketing, sampling survey, AGIL scheme

## Abstract

Globalization intensified competitiveness among agribusinesses worldwide in recent years. The European Commission focused on enhancing sustainable agriculture and food products’ territorial uniqueness for competing in the international market. The Mediterranean diet (MD) is a model of feeding and lifestyle belonging to the ancient Mediterranean culture, which also embodies a sustainable food system. Therefore, in 2010, UNESCO recognized the MD as Intangible Cultural Heritage of Humanity, and Sicily (southern Italy) is its official physical site. Despite its worldwide fame, the notion of the MD runs the risk of being mystified because it is described most often as something that does not correspond to what it is holistically. The aim of this study is to know the market demand of the MD by Italian people and foreign tourists in Sicily and the level of knowledge of the MD by users, both consumers and experts. A survey at top Sicilian traditional restaurants with owners/chefs and their clients was carried out. The study provided an in-depth understanding of the current lack of knowledge about the holistic meaning of the MD. The study highlights the desirability of integrated science–policy actions (also for communication) and proposes a vertical communication system to revive and direct the MD demand toward its holistic model.

## 1. Introduction

Globalization has triggered a stronger competitiveness among agrifood enterprises worldwide during recent years [1,2]. The interdependence of the world’s economies, cultures, and populations has led the EU Common Agricultural Policy and agribusinesses to exploit the uniqueness characteristics of food given by territorial differences for obtaining a competitive advantage in the international market [3]. These elements of uniqueness unquestionably characterize agri-food products that are nothing more than “the product” of the territory. Irreproducibility is their main value, which in turn connotes intrinsic quality [4,5,6]. Specific territories, thus, can be identified in such food products that are an expression of them. In Italy, there is a strong identity link between territories and their food products—just think of wines such as Brunello di Montalcino DOCG (Denomination of Controlled and Guaranteed Origin) or Parmigiano Reggiano cheese DOP (Denomination of Protected Origin)—because of the strong connection between food and the territory’s history, culture, and economic development [6,7]. Moreover, the territorial pedoclimatic characteristics have a further key role in characterizing unquestionably agricultural food products and wines, particularly in Sicily [8]. The impossibility of reproducing certain food products in other parts of the world due to the irreproducibility of the pedoclimatic and cultural elements that generated them is precisely the strength of such products, their greatest value, which connotes the intrinsic qualities and traits and can be defined as the brand-land value (and Country of Origin effect or Made-in effect) [9]. The struggle of Italian and European agribusinesses for survival in the global marketplace has, therefore, territorial specificities, product identification, denominations of origin, and innovation within the tradition as elements of competitive strength. The specificities of Italian agricultural production are the result of centuries of experimentation by farmers, which has allowed them to select species, varieties, and animal breeds that, over time, have become characteristic of traditional regional productions [9,10]. 

Territorial specificities with local knowledge and culture represent an economic resource with value of innovation for farmers in the EU [11,12]. At the same time, the agriculture sector in the global context is considered to be one of the main causes of climate change and world pollution. In fact, food production is responsible for up to 30% of global greenhouse gas emissions and around 70% of freshwater use [13]. Certainly, feeding the global population is an issue and doing it with healthy food is a concern taken increasingly into consideration by governments. In 2022, during the third “World Conference on the Mediterranean Diet”, participants wanted to focus on the importance of the Mediterranean region for the growing interdependent challenges that are undermining the sustainability of food systems and that are negatively impacting their populations and natural resources [14]. During the same conference, the “inegalitarian drift” in the current relations between Northern Mediterranean countries and Southern–Eastern ones was analyzed. According to the Conference, this drift exacerbates the already existing difficulties in political relations due to the actual great heterogeneity between countries and due to an ever-growing gap between developed economies and those less developed (a gap that may generate economic, social and cultural conflicts) [14]. Finally, the COVID-19 pandemic and the disruptions of imports from Ukraine and Russia further aggravated the situation of a region in a “post nutrition transition state” [14,15]. The prevalence of undernutrition (wasting, stunting, underweight) and micronutrient deficiencies in some territories is overshadowed by the incidence of overweightness, obesity and diet-related chronic non-communicable diseases with undesirable impacts not only on the health and related public expenditure, but also on the cultural, social, economic, and environmental sustainability dimensions [15,16,17].

Accelerated climate change has further exacerbated existing environmental problems in the region that are caused by the combination of changes in drought and desertification processes, increasing pollution, and declining biodiversity [18]. Therefore, the Mediterranean region is now facing four big challenges: environmental, economic, socio-cultural, and health-nutritional challenges. 

The Mediterranean Diet (MD) pattern has been shown to have a better ecological footprint than current dietary habits in industrialized countries, particularly when compared to the Western dietary pattern [19,20,21]. This is mainly due to the higher consumption of local and in-season plant-derived foods and the lower consumption of animal products. The MD is not only a healthy dietary pattern inspired by the traditional eating styles of people living in the countries bordering the Mediterranean Sea (at the crossroads between Africa, Asia, and Europe) [10,14,17], it is an intangible and sustainable food culture, a “way of life” that also embraces important physical, socio-cultural, economic, and environmental benefits [14]. 

The conference, as part of the UN Food Systems Summit movement for change, will accommodate multiple perspectives to improve the sustainability and resilience of food systems in the Mediterranean [14]. In this complex scenario, the MD was presented as part of the solution. In fact, it was observed that dietary patterns like the MD are healthier and, at the same time, exert a lower impact (a better ecological footprint) on the environment than current dietary habits in industrialized countries [22,23,24,25]. This is mainly due to the higher consumption of local and in-season plant-derived foods and a lower consumption of animal products [23,24,25]. Particularly, the MD was defined by the Food and Agriculture Organization (FAO) as one of the most sustainable food regimes on the planet [22]. Therefore, a broader adherence to this dietary model would make a significant contribution to the greater sustainability of the food system (from producer to consumer), with a myriad of benefits for human and planetary well-being [23,24,25]. Moreover, the MD could play an important strategic role as food-associated habits for the Food Policies in the Mediterranean area, both for the metropolitan cities [26] and for the rural areas [26,27]. Nowadays, current dietary styles in Mediterranean countries are departing from the traditional MD pattern to new adaptions of Western and other food cultures with regard to quantities and proportions of food groups [25,28]. Particularly, regarding the MD, the erosion of the MD heritage from the loss of its adherence among Mediterranean populations was considered very important during the conference, as it has undesirable impacts not only on health, but also on social, cultural, economic, and environmental trends in the Mediterranean region [18,25]. This can be explained by the fact that the MD is not only a model of cultural food choices, cooking methods, meal patterns, and, more broadly, a lifestyle, but it is also a sustainable framework that attenuates the environmental pressure of food production and consumption [29,30]. Transitions from actual food consumption patterns (that in most European Mediterranean countries evolved parallelly to economic growth) towards a traditional MD model [28] require substantial changes in consumers’ values, education, and choices. This is the challenge not only for current policy makers, but also for academics and scholars who study the Mediterranean diet from different perspectives.

### 1.1. Historical Overview of the Mediterranean Diet and Updating of the Mediterranean Diet Pyramid

The term “Mediterranean Diet” was coined in the mid-1970s in the United States by Ancel Keys (1904–2004), one of the most prominent scientists of our time, and his wife Margaret Haney [29,30]. They became effective promoters of a healthy lifestyle and food consumption model. In their publications, the two scientists were the first ones in the English-speaking world, which proved the value of the Mediterranean diet (from the Greek δίαιτα = *diaita*, alias “way of life” [31]) basing on their scientific research combined with culinary art. 

The first “Mediterranean Diet Pyramid” (MDP) was elaborated on by the U.S. Department of Agriculture, basing it on the studies conducted by Mr. and Mrs. Keys (who also experienced for themselves the positive effects of the Mediterranean lifestyle, reaching a considerable age) [32]. It is thanks to their studies that, later, the World Health Organization (WSO) was encouraged to promote the MD to improve global health and to use the MDP as a pedagogical tool exemplifying its basic rules. 

In 2010, the MD was recognized by the United Nations Educational, Scientific, and Cultural Organization (UNESCO) as an Intangible Cultural Heritage of Humanity [33]. This important recognition was given by providing the following definition of the MD. From the meaning of the ancient Greek word, δίαιτα = *diaita*, alias “way of life and lifestyle”, the MD is a set of skills, knowledge, practices, and traditions from the landscape to the table. It is around the table that words play an important role in the description, transmission, and celebration of this principle. The MD, is the result of constant sharing, nurtured by internal synergies but also by external inputs, a place of fusion of traditions, innovations and creativity applied for centuries. It represents the people’s lifestyle in countries of the Mediterranean basin” [33]. According to the UNESCO’s recognition, the MD, thus, “constitutes a set of skills, knowledge, practices, and traditions ranging from the landscape to the table; it represents a succession of social practices and policies that have transformed simple food into an identity symbol and a community tool of aggregation” [33]. In summary, it is a real way of life. 

In 2011, the FAO awarded the MD, defining it as “one of the most sustainable food regimes on the planet” [22]. With this important award, the MD was also identified as a “strategic asset for the development of the economy, peace, and cohesion among the peoples of the Mediterranean basin” [22]. In fact, during a FAO/CHIEAM workshop, the sustainability of the MD model in the Mediterranean region was assessed for the first time. Following this award, the Mediterranean diet was also called to respond to the new sustainability challenges of that area thanks to its four dimensions: health and nutrition, environment and biodiversity, economic, and socio-cultural factors [34].

In 2014, the International Mediterranean Food Foundation (IFMeD) was founded with the aim to embrace the multidisciplinary knowledge and expertise of the MD and to revalorize and enhance the MD as a healthy and sustainable model of lifestyle and an intangible cultural heritage of humanity [35]. In 2015, during the Milan EXPO, the Med Diet 4.0 framework was presented for the first time and many cities, also including Palermo (capital of Sicily region), signed a memorandum of understanding to create a network of metropolitan cities committed to the promotion and implementation of sustainable food policies [36].

Moreover, the “Milan EXPO 2015” (World Exposition Milan 2015, Italy), dedicated a week to the Mediterranean diet [36], and some important ideas and reflections were set out in the “Milan Charter” to also recognize the value of the Mediterranean diet in terms of lifestyle [37]: “Eating is not a mere satisfaction of physiological needs, but a convivial occasion, of sharing, of intergenerational, intercultural and interreligious dialogue”. The considerations of the Milan Charter are very important because they consider the diet a social lifestyle as well. 

The MDP was revised many times during the last 50 years due to changes in people’s lifestyles and new scientific findings in the medical sector [38]. For example, refined cereals (such as pasta, white bread, potato) have been considered foods to be consumed moderately. This is because whole grains have a lower glycemic impact than refined cereals and are healthier for human health. In addition, only animal fats were indicated to be consumed occasionally. Contrarily, vegetable fats (such as olive oil) were moved to the base as products to be consumed daily [38,39]. 

In 2020, during the third world conference on the MD, the MD pyramid was officially revised by the Board of the “International Foundation on Mediterranean Diet” (IFMeD) [23]. This updated animal protein sources are positioned to suggest a lower frequency of consumption and contribution to the total intake, and the top of this pyramid presents both sugar and animal protein foods (e.g., pastries, sweets, and red and processed meat), that should be consumed only occasionally [23]. Moreover, this new pyramid clearly and incontrovertibly introduces the elements underlying the Mediterranean diet, namely those pertaining to the regular and daily practice of mild/medium physical activity and the “conviviality” during meals. This conviviality implies meals are eaten slowly, placing “slow food” clearly in antithesis with so-called “fast food” because of proven beneficial implications for mental and physical health [23]. However, the conviviality during meals was precisely a typical feature of the ancient lifestyle of the Mediterranean peoples.

**Figure 1:**
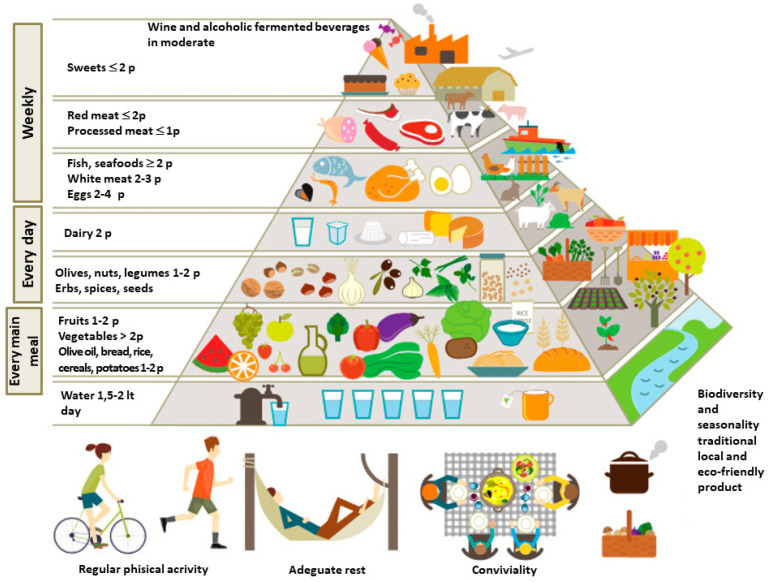
Authors’ editing of the sustainable Mediterranean diet pyramid developed by Lluis Serra-Maiem et al., 2020 and other authors [23,38,39,40,41,42].

The most innovative element of this updated pyramid, alongside the repositioning of certain foods and the consequent change in their prescribed frequency of consumption, is the visual representation of the third dimension of the MD, namely that of sustainability and biodiversity and the impact that each type of food has on the environment [23]. In this way, the pyramid is a tool to describe in a clear and understandable way how the foods favored by the MD have a very low environmental impact; this environmental impact goes gradually increasing as one goes up the pyramid in correspondence to the other foods that are less recommended or to be avoided (Figure 1).

### 1.2. Mediterranean Diet and Biodiversity in Sicily

Sicily (island of southern Italy) was historically the meeting area of ancient millenary civilizations coming from a variety of countries with marked environmental, cultural, social, economic, and political differences [43]. For this reason, this island was one of the first receivers of plants and animals of Middle Eastern origin and had the aptitude to include them in the first agricultural–livestock process of inclusion and fusion, which provided extraordinary opportunities for this land and its inhabitants [43,44]. 

It was the instinct of locals, especially women, that favored the selection of plants to be grown, fruit to be consumed and animals to be raised, recognizing their beneficial effects on their men children [43]. To sanction this, the UNESCO in the MD definition specifies the following [33]: “for centuries women have played a fundamental role in the transmission of knowledge of the MD as they take care of family members and acquaintances by preparing both daily and festive food and pass on their culinary secrets to children and grandchildren, making festive banquets an authentic celebration of life. Moreover, when rationality took over, it was useful to introduce new arrivals, including products that arrived in Europe after the discovery of America, such as tomato, potato, corn and many others, the use of which did not subvert previous habits, but only integrated them. This has allowed maintaining the fundamental character of the MD. These consumptions, in the Mediterranean area, have imposed themselves spontaneously and today find many paths open to the rest of the world” [33]. 

Today in Sicily, there are over 2700 specific and intraspecific vegetal species, of which about 400 are endemic (that is, present only on the island) [45]. In the dry inland area, arable crops (cereals-hard wheat, legumes, and winter vegetables), dried fruits (almond, hazelnut, and pistachio), olive trees, and vines were grown for ages. In the more irrigated area, citrus, vegetables, and fresh fruit. Cattle, sheep, and goats are raised in the wild and semi-wild state in natural pastures. Edible and aromatic wild herbs are found scattered throughout Sicily and especially in inland hill and mountain areas. Fishing was practiced from the many ports in Sicilian coastal areas. This taxonomic variety is accompanied by a high genetic variability of many of these species that go to make up the enormous biodiversity of this region. Such biodiversity involves as much in fruit plants, among them especially citrus, but especially in horticulture and vegetables and aromatic plants. The genetic variety to which many species have been subjected over time derives from their ability of adaptation to the different pedoclimatic Sicilian environments (i.e., humid, temperate, arid, and drought-prone depending on the territorial zone). The different genotypes of individual species have therefore over time differentiated with different flowering and ripening epochs, adapting to different climatic zones, because it allows for the cultivation of several native and non-native species. Over time, all this has allowed for the availability of products shaped by the different territorial areas, which, in turn, characterized local communities’ gastronomic traditional preparations over several decades. 

Examples of this are in the horticultural field the artichoke (Cynara cardunculus), whose rich heritage of varieties is now at great risk of genetic erosion, given the increasing spread of an exotic germplasm characterized by high productive capacity and commodity uniformity and the consequent loss of the rich heritage of Sicilian varieties with different characteristics and uniqueness in terms of adaptability, rusticity, and commodity and organoleptic typicality [45]. Further reference can be made to species of the Brassica genus, such as cauliflower, broccoli cabbage, kohlrabi, and leaf cabbage, whose original genetic heritage is in danger of being lost due to crosses with hybrids aimed to make them more productive [45]. As many as 96 different genotypes of fava bean (*Vicia faba* L.) found in different localities of central–eastern Sicily have been classified in the last 30 years, to which must be added those of the chickpea (*Cicer arietinum* L.), bean (*Phaseolus vulgaris* L.), lentil (*Lens culinaris* Medik.), and chickling vetch (*Lathyrus sativus* L.), which count a total of 129 different genotypes. Sicily, since the time of the ancient Romans, has been regarded as one of the granaries of the Mediterranean, and over the centuries, cereal farming has been the main source of livelihood in several inland areas of the island. This has led to the spread over time of a huge variety of cereals and wheat (Triticum durum). In recent years, however, some of the ancient grains (Perciasacchi, Russello, Timilia) have been relaunched for their organoleptic qualities. Finally, also worth mentioning are the numerous genotypes of olive trees (*Olea europea* var. *europea* L.) [46]. In 2002, a large olive germplasm collection was established in Sicily, which today contains eight well-known and extensively grown cultivars, 17 minor or neglected cultivars, and 122 native genotypes [46,47]. The biodiversity present in the Sicilian territory also concerns cattle farms with native breeds (Modicana, Cinisara) for meat and milk, pig farms (Nebrodi black swine), and goat farms (Girgentana and other Slow Food presidia [48]). 

### 1.3. Mediterranean Diet, Gastronomy, and Tourism in Sicily

Nowadays, Sicily is the Italian region with the greatest number of different plant species [45]. Certainly, Sicilian food products’ richness and variety is due to its location in the center of the Mediterranean Sea, which has fostered the meeting and fusion of peoples and cultures with their agro-food products. This enormous biodiversity has allowed for the development over time of a gastronomic culture with the structuring of preparations closely linked both to the seasonality of the various genotypes of the different plant species and to the rural territories and individual traditions connected to them. In addition, the favorable environmental conditions of the largest island of the Mediterranean Sea allowed for the production of agricultural food products and dishes that were disseminated and appreciated around the Mediterranean area [49,50]. These circumstances caused a strong qualitative boost that brought food and local cuisine to high quality standards at that time [43,44,49,50]. Sicilian gastronomy developed over the centuries, with many dishes made with recipes that include the use of different products following their seasonality and the influence of the neighboring Mediterranean countries. This made Sicily one of the first fusion food laboratories for importance, and it resulted in a unique Mediterranean territorial cuisine. 

The loss and flattening of the enormous Sicilian biodiversity due to the use of more productive and profitable varieties and genotypes caused the loss of gastronomic traditions in the different rural environments of the island. Similarly, this happened at different extents in all the Mediterranean territories. However, the relationship that links the food model with the places of production contributes to determining the identity characters of landscapes, territories, and communities whose food represents the connective element [9,51,52,53]. Even today, the traditional Italian, and particularly the Sicilian, “meal” represents the symbol of welcome, Mediterranean inclusion, and social integration.

Experiencing foods and territory is nowadays one of the most popular choices for spending leisure time by tourists, visitors, or simply users of a rural area [9,51,54]. Food thus becomes an opportunity to “experience” the territory through a direct involvement of the user in the food and wine, cultural, and environmental specialties that characterize the places of origin of the products [54]. Moreover, food and wine today are also a social trend and fashion phenomenon. More and more people talk about food and also share experiences via social networks [55], not only in everyday life, but also during the journey [55]. The food and wine tourism sector is growing steadily in Italy [54]. Particularly, for the 63% of tourists who arrive in Italy, food and wine are two of the main reasons for choosing a destination for a trip [56,57,58,59,60,61,62]. Italy, in fact, is the first place in Europe for the popularity of the cuisine to be important among European vacationers [63,64].

Following this ascertained relationship between agriculture, food traditions, food-territory identity, Mediterranean diet, and food and wine tourism [64], the Sicilian regional Parliament, in 2022, issued a regional law to formalize the role of the MD as a fundamental element of Sicilian local identity [65]. The law “Recognition and promotion of the Mediterranean Diet” (DL no. 896-547/A) aimed at promoting public and private initiatives to increase consumer awareness of the benefits and sustainability of the MD and, at the same time, enhance it for “the protection of the historical and cultural heritage, that is expression of the identity of the Sicilian territory”. While on the one hand, this law aims to enhance and promote the Mediterranean diet model at the local level, on the other hand, it takes advantage of the recognition of the MD by the UNESCO to also make the MD an additional tourist attraction for Sicily, which is its official material site. This law also promotes sustainable agriculture and local food production (sustainable food system) and encourages environmental protection actions by farmers. Moreover, it promotes the healthy lifestyle of the MD pattern and the culture of respect for Mediterranean lands and cultures. The new law also funds the creation of a multifunctional MedDiet museum and the opening of tourism routes to facilitate tourists’ discovery of high-quality food local products and their diversity among different regions. In addition, this law identified the day 21 March as the “Regional Day of the Mediterranean Diet” in explicit reference to the UNESCO’s award. Educational programs on the MD for schools and universities are also sponsored.

Alongside this law, also in 2022, the planning of the first “World Park of Mediterranean Lifestyle” or also “First Polycentric and Widespread World Park of Mediterranean Lifestyle” started in Sicily [66]. This project is a vast area territorial development project that, thanks to a community pact, manages to involve about three hundred public, private, and social partners, distributed in all the region’s areas with the aim to promote the Mediterranean lifestyle community and the experiential tourism. According to the park’s inventors, a complex work of systemization of all cultural tourism attractors in Sicily should be done by spreading the Mediterranean model through the anthropological, educational, ecological, economic, food, health, and sport dimensions. 

### 1.4. Literature Advances and Gaps, Objectives of the Study, and Research Questions 

The Mediterranean culture teaches us that it is possible to combine health and happiness, careful eating and the joy of companionship, a sense of responsibility and conviviality, and a desire to cook and to set a beautiful and well-kept table [64,65]. The table thus becomes a memory of who we are, a history of our family, a dialogue with the people we care about most, socialization, and at the same time a guarantee of good health and longevity. Moreover, the economic activities that are the basis of this model are respecting the environment and guaranteed human-friendly resource management and care for nature; this is the reason why the UNESCO wanted to give this important recognition to the MD [33]. 

The Mediterranean diet has a high value for its nutritional, health-sanitary, social, and environmental scopes. The Sicilian region is institutionally recognized as the material home of the Mediterranean diet because of its strategic position in the Mediterranean and its unquestionable characteristics of biodiversity and culinary experimentation/fusion among the Mediterranean. Italian food has always been a flagship of the country’s economy and a major tourist attraction for foreign tourists from European and extra-European countries. The Sicilian region has recently recognized with a regional law the importance of enhancing the Mediterranean diet not only as a healthy and sustainable food system, but also as a tourist attraction because of the close link between cultural heritage and tourism [65]. 

Several studies have been conducted so far regarding the MD and its value related to its well-documented healthy benefits for human health and the environment [67]. Nevertheless, as far as we know, very few studies investigated the level of correct knowledge and awareness of the population about the Mediterranean diet in its holistic mode. Despite the fact that there are some authors who observed that the MD as a dietary regime is widely followed among the Sicilian population [68], many doubts about its mystification arise considering that there is no evidence of the level knowledge on the part of the population (consumers, farmers, food tourists, or restaurant industry experts) regarding the content of the Mediterranean diet model [69]. In fact, other authors investigating the adherence to the traditional Mediterranean diet [70] in a population in the south of Italy [71] conclude by suggesting improvement in people’s knowledge about food and nutrition, taking into consideration beliefs embedded in their family, tradition, and religion. Knowledge transfer within information systems is considered to be an important topic for researchers, policy makers, and practitioners [72]. The theoretical model of some studies proposes that the source’s capability, credibility, and extent of communication will play an important role in determining the extent and quality of knowledge transferred to recipients [72]. 

The study tried to fill the gap in the literature providing a first initial picture of the current level of information and knowledge of people about the MD starting from Sicilians and Italian/foreign consumers. The aim was to have the first information about the actual demand of the Mediterranean diet among people and, thus, their level of knowledge about the MD pattern in its holistic meaning. It also possibly proposes a suitable communication/information model resulting from the elaboration and interpretation of the study’s results. It was decided to start from Sicilian people and tourists visiting Sicily because Sicily appeared very suitable considering all the scientific evidence and the circumstances extensively described in the previous pages of this section.

For this study, the clients of the top traditional restaurants in the Sicilian region were surveyed. The basic assumption was to hypothesize that if a tourist arrives in Sicily and asks for the MD wanting to experience it, he or she would go to one of the typical traditional restaurants and try to get to know something more by tasting the typical dishes and talking to the chef. Moreover, it is well known that chefs are deemed “ambassadors” of a territory since in preparing the dishes, they must know their history (culture) and the origin (agriculture-land) of the raw materials. Therefore, for the purpose of the completeness of the study, it seemed appropriate to also interview the chefs of these restaurants so as to also know their level of information, knowledge, expertise, and awareness of the MD model. In summary, this study tried to answer the following research questions:

**RQ1:**  *What is the actual level of information about the MD and its adoption among Italians and foreigners? What are the communication channels through which information is retrieved? Customers of traditional restaurants ask to eat a “MD meal”? [Considering traditional restaurant (e.g., restaurant, trattoria, pizzeria, bistrot, tavern), this study focused on restaurants, trattorie, and taverns. A traditional restaurant has the characteristic of possessing knowledge, skills, customs, and practices of a culture or group that conveys the preparation of special recipes or preparation methods handed down from generation to generation. It is characterized by personalized service at the table, relatively long waiting times due to the choice of a la carte food, and a higher price compared to other forms of catering. Moreover, a traditional restaurant offers a national and regional cuisine with a strong link to local products. The “typical” restaurant has, in addition, a connotation for cuisine, furniture, and service very linked to the place].*

**RQ2:**  *What is (and what should be) the role of food service experts, such as chefs/maître de sale/restaurant owners, and their knowledge about the MD model (lifestyle and sustainable food system) for its promotion?*

**RQ3:**  *What are the main media used to acquire information regarding the MD? Is the MD pattern communicated effectively among the population? If not, what could be a possible model of communication for locals and tourists to avoid the mystification of the MD?*

## 2. Materials and Methods

### 2.1. Methodological Approach

#### 2.1.1. Netnographic Analysis

Netnographic analysis comes from the combination of “Internet” analysis and “Ethnographic” analysis. In 1995, Robert V. Kozinets coined the term “Netnography”, which refers to ethnographic research on people’s online interactions. It is a specific methodology used to identify communication patterns and behaviors in groups and subgroups of online cultures or communities [73]. With the increased use of social media, Netnography has spread to the fields of marketing, business, and management. It has been useful for both sociological and marketing objectives because it has appeared to be well suited to the study of the culture of the online consumer. Today, social media companies, such as Facebook, Twitter, and Instagram, are increasingly becoming a valuable marketing source of public information. In 2018, Kozinets established four types of Netnography: “auto Netnography”, “symbolic Netnography”, “digital Netnography”, and “humanistic Netnography” [73]. Auto Netnography is focused on autobiographical aspects (e.g., personal thoughts when participating in social network conversations). Symbolic Netnography was derived from the need to provide decision aids for managers with the goal of identifying consumers [73]. Digital Netnography is achieved through some techniques of analyzing statistical data. It focuses on incorporating a social media data set to identify patterns and links on members’ cultural elements. The fourth and final typology is humanistic Netnography. It focuses on critical research and critical theory, guided by social issues of great importance. For this study, we applied digital Netnography.

For this study, initially, the types of content that resulted when searching the keyword “Mediterranean Diet” on the internet and on social networks were observed with the aim to know how the MD was “discussed” in the social networks and Internet and the main topics of discussion among experts and peers or among peers. To do so, the keyword “Mediterranean diet” (in the English language) was searched on Google and on the most used social networks (specifically Instagram). Around 7,000,000 results emerged from the Internet and social networks. The first 70 websites and social media profiles were observed in order of findings, considering: number of views for websites and number of followers for Instagram profiles. The contents were analyzed by authors using the Netnographic analysis [73]. Specifically, for each single web site or post, the following data were extracted and analyzed: publishing date, number of articles/posts, topic and information about the MD (characteristics and its benefits for the human health and for the environment), types of people involved (experts or users), type of experts, and user.

#### 2.1.2. Sampling Survey

After this preliminary analysis, a sample of chefs/owners/directors (experts) of traditional restaurants in Sicily was observed (AN1), and a sample of people, among their customers (AN2) both Italians and foreigners, was interviewed. 

AN 1: For the survey with experts the top Sicilian traditional restaurants were identified based on a study on TripAdvisor. The restaurants/taverns with the highest score of references and reviews (expressed in stars) received by clients were identified [74]. Of the top 90 restaurants selected for the study (statistical universe), 7 did not want to cooperate, 13 were not available, and 10 did not submit the filled questionnaire. Therefore, the final number of restaurants identified was equal to 60. Of these restaurants, the chefs/owners/directors were interviewed. It was chosen to survey these experts because they are those in a traditional restaurant who tell customers about the dishes offered and the raw material used and the history of the dish with its links with the local territory. Thus, they are important conveyors of information and knowledge about food.

AN 2: For the survey with people, a sample Italians and foreign clients of the selected restaurants was randomly selected [74,75,76]. The randomization of customers was carried out to understand on average the people’s consumption habits and lifestyles and their information about the MD in its holistic meaning. Restaurant customers were chosen because it was going to identify a sample of people interested in traditional local cuisine and Mediterranean dietary pattern. This segment of consumers allowed us to focus on a segment of people possibly interested in experiencing the MD, both Italians (particularly Sicilians) and foreigners, even only from a culinary point of view and not necessarily as a lifestyle. For interviews with restaurant customers, restaurateurs were asked to ask their customers if they were willing to participate in a study on the MD, and if so, they were asked to fill in a questionnaire accessible via a link to a digital form. In this way they could participate in the survey at any time, even later, and using smartphones, tablets, or computers [49]. The appropriate sample size was calculated with a confidence interval where *p* = q = 95% (ε = 5%) and a sampling error in the range between 3% and 4% (which implied a sample size from 1.111 to 625 participants) [49]. In this case, the survey was stopped at 657 received questionnaires. After a prior elaboration of questionnaires aimed to obtain a balanced sample, the valid questionnaires used for this study resulted in 650.

Both for AN1 and AN2, participants were asked to complete a questionnaire on the MD. They were informed about the academic research in writing and asked to consent to participating in the research inside the questionnaire. In addition, all participants were told that the research was voluntary, and they could withdraw at any time. All participants had to declare they were healthy and without any medical condition that imposed a particular dietary regime. The interviews were carried out from April to September 2021. Specific written instructions were given at the beginning of the questionnaires about how to fill the questionnaire. All the procedures performed in this study were anonymous.

#### 2.1.3. AGIL Analysis

The AGIL method (originated from Talcott Parsons, 1961) [77] is a model used to figure out and interpret the dimensions of “communication”, one of the principal phenomena of social interactions and relationships. AGIL analysis is a very successful multidimensional method that can be used to investigate complex subjects, such as communication, and to measure its different aspects. Parsons T. suggested this qualitative–quantitative model, adapting it for each type of society and its system, and nowadays, it is frequently applied for the rapidity of obtaining results and also for its relatively low cost [77]. Since this multidimensional methodology is so flexible, it was also used for other types of research in the marketing field. Since it improved recently and it was also applied to some food product studies, it was considered appropriate for communication model of this study. As the original Parsons scheme, the four dimensions suggested by the acronym AGIL were identified: Adaption (A); Goal attainment (G); Integration (I); and Latent pattern maintenance (L) [63]. In detail, the “Adaption” (A) dimension follows the principle of optimizing resources and means that it has a persuasive function through which communication finds itself operating in social structures (market, mass media, digital media, and institutions) and satisfying multiple and different objectives. The “Goal attainment” (G) dimension indicates the cognitive dimension that operates through the distribution function of disseminating information; it follows the principle of realization of the dissemination action. The “Integration” (I) dimension concerns the norms of community in which the communication is carried out and follows the principle of compliance with tradition and notions shared by the group. Here, instead, communication was considered from the point of view of the relevance of participation in community events. The last one is the “Latent patterns maintenance” (L) dimension, which relates to communication as an expressive form of the identity of the individual or collective social actors [63]. In this study, it was applied to provide a deep description of the characteristics of each step of the communication model.

### 2.2. Questionnaires

The questionnaires were tested by a panel of experts to check for consistency and coherence. Subsequently, we carried out a pre-test of the questionnaire with the method of self-compilation with assisted interviews to a small sample of individuals extracted at random within the university campus with the aim of verifying the clarity of exposition and the unambiguity of the questions, the simplicity of the language used, and the suitability of the type of response of each question in order to simplify and speed up the process of response from the respondents. After the test, the questionnaire was created with Google Modules and made accessible through links. This made it possible to disseminate it via various social media (private accounts and public pages of Facebook groups) and messaging applications (WhatsApp and Messenger) and by emails for restaurants.

AN 1: The questionnaire given to the owners or directors of the activities that provide the catering service was given to understand how many companies exploit the MD as a local resource and how it is really known by people in the sector. It consisted of 18 questions asking about the type of cuisine offered to customers (if they used raw materials from organic farming, seasonal products, 0 km products, products of Italian and/or local origin), and about the MD.

AN 2: The questionnaire for customers was used to understand the eating habits of the interviewees and how much the MD was known, and it was structured in two languages according to the origin of the respondents. The questions in both formats were identical except for the provenience. The one in Italian required the region of origin while the one in English required the country of origin. It contained 31 questions with a closed answer. The first part included 11 demographic questions about gender, region/country of residence, age, height, weight, level of education, composition of household, current occupation, hobby, and type and time of physical activity. 

The second part included additional 11 questions asking general information about the eating habits of the interviewee and other activities usually practiced during spare time or vacation. The third part as well as the last included 9 questions that explicitly asked respondents about their knowledge of the MD.

## 3. Results

### 3.1. Preliminary Netnographic Analysis

With the Netnographic analysis on websites and social networks, five main topics of discussion about the MD were highlighted, which we defined as “key-findings” (KF) as in previous studies [8]. The first was (KF1) “Benefits of the MD for human health” (31.9% of contents found); generally, this topic was discussed or authored by doctors, biologists, nutritionists, and dietitians. The second topic was (KF2) “Procedures to prepare dishes belonging to the MD” (23.8% of contents), covered mainly by chefs or people without any title. The third key finding was (KF3) “Information about events, congresses, seminars, and other opportunities to talk about the MD” (20.2% of contents) by organizations and associations or public institutions, scholars, etc. The fourth topic was (KF4) “Benefit of a health nutrition, with reference to the MD style” (about 18.4%) with discussions among common users through blogs or social networks (particularly Instagram) with questions and answers among the users themselves with the aim to learn about the benefits of a proper nutrition (peer-to-peer communication). Finally, the fifth key finding was (KF5) “Recommendations to combine a healthy diet like the MD with sports and physical activity” (“*mens sana in corpore sano*”) (about 5.7% of contents). Generally, this topic was found at web pages of sportsmen/sportswomen and fitness coaches or social media profiles of all types of people who suggested, besides a good workout, a healthy diet such as the MD are more balanced both physically and psychologically or discussed their experience with this. This Netnographic study showed that, so far in the social networks and Internet, the MD is mainly considered to be only a gastronomic and dietary regime effective for health. In addition, the information about the characteristics of the consumption proportions of recommended food doses according to the MD was not always correct and accurate except in some cases when provided by doctors or experts. In general, users appeared to be interested but unaware of what the MD is. Moreover, “experts” in the blogs, Internet sites, and Instagram pages also appeared lacking a correct knowledge of the MD. Therefore, it was possible to conclude that information conveyed through social networks about the MD is mystified. 

### 3.2. Analysis of Restaurants’ Owners

As shown in Table 1, regarding the variable “Type of cuisine offered”, 80% of the participants said they offered “local traditional” cuisine; 11.7% said they offered a type of local organic cuisine, and 8.3% offered a cuisine specialized in fish. No one said they offered only vegetarian cuisine or vegan or halal kosher (The term “Halal Kasher” or Kosher refers to religious certifications that ensure respect for Islamic or Jewish precepts during the slaughter and processing of food).

Regarding the origin of raw materials used to prepare the dishes, 100% of respondents declared they use local, regional, or, at least, Italian products. The restaurants’ owners also declared that their customers were “highly interested in the consumption of local products” (high 61.7%) and “very interested” (good 33.3%) (Table 1). In addition, almost all the restaurants’ owners (75%) declared they buy 0-km food products for preparation of the dishes, and 96.7% prefer seasoned products. This highlights that local products for meal preparation are considered of relevant importance.

**Figure 2:**
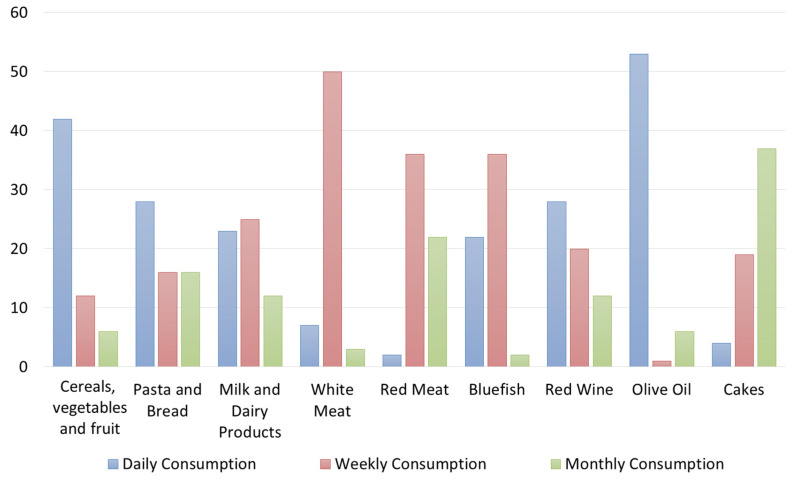
Own processing of the results regarding restaurateurs’ knowledge of the frequency of food consumption provided by the Mediterranean Diet Pyramid.

Regarding the level of knowledge of the restaurant owners/managers/chefs about the meaning of the MD, 71.7% of respondents self-reported to have basic knowledge, 18.3% declared themselves experts about the MD, and 10% said they have just sometimes heard about the principles of the MD. However, 23% of respondents declared they “always” offer a meal organized according to the MD, 45% said “often”, 22% “sometime”, and the remaining “10%” declared they offer it “rarely” or “never” (Table 1). These results highlight that restaurateurs generally propose and explain the dishes offered within their menus, but they do not explain to customers who ask about the MD that, in addition to the foods, proportions and portions of them in a meal (consisting of several courses) also uniquely characterize the MD style (Table 1). According to the data, however, only 0.3% of respondents said that their customers always asked for menus based on the MD, 13.4% said that this request happens “often”, 40% of respondents declared that “sometimes” customers have asked to consume menus following the MD, 15% declared that this has happened rarely, and 28.3% declared that nobody ever requested the MD menus. Almost less than 50% of the sample said that the MD is a UNESCO Intangible Cultural Heritage of Humanity (Table 1). However, 38.3% of respondents consider the MD a local intangible resource to enhance the Sicilian territory or the Italian food products. Regarding the level of knowledge of restaurateurs regarding the foods’ frequencies of consumption indicated in the MD pyramid (Figure 2), the results show that their answers are partially incorrect since they declared a daily consumption of vegetables, fruits, tubers, and cereals and of olive oil. Pasta and bread were declared to be consumed also weekly and monthly, similarly to the red wine.

Contrarily, pasta and bread should also be consumed daily (at each main meal in a limited dose) like fruits and vegetables, albeit in larger doses. 

Another inaccuracy relates to the consumption of white meat and milk/dairy products, which they declare should be consumed daily, and instead, according to the MDP, they should be consumed weekly (Figure 2). This is an example of new adaptions of Western and other food cultures with regard to quantities and proportions of food groups and of a departure from the traditional Mediterranean dietary model. Another mistake was made for wine, which, although it represents a traditional food product of the countries of the Mediterranean basin, it must be consumed at most weekly in minimal doses (Figure 2) because it leads to the development of diseases, such as diabetes and obesity. Moreover, the respondents declared that desserts, which are foods that close a classical Italian meal, can be consumed weekly or even daily. Instead, the MD Pyramid suggests consuming a maximum of two portions of sweets and desserts per week.

**Figure 3:**
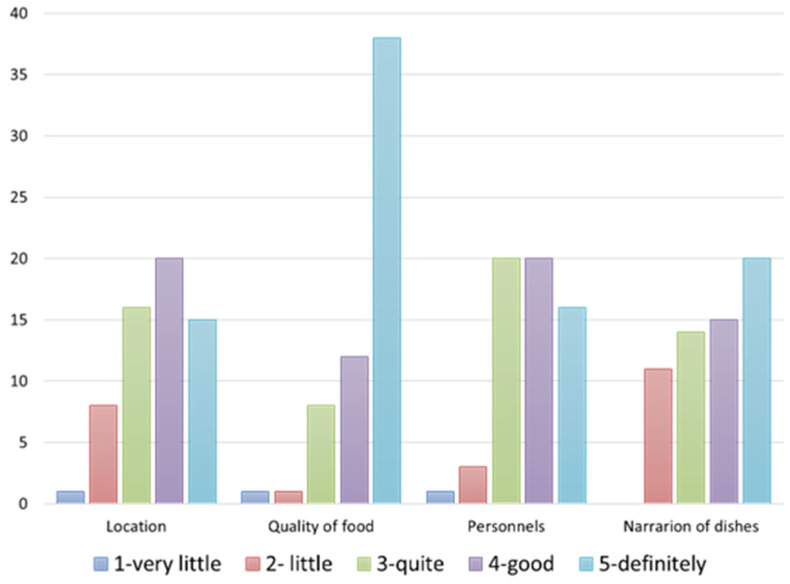
Own processing of the results regarding restaurants’ strengths according to restaurant’s owners.

Respondents were asked to assign a level of importance (from 1 to 5) to certain variables they considered as a restaurant’s strengths. Restaurants’ owners declared that the major strength is the “quality of food” offered to their customers (70%). However, “narration of the dishes, stories, traditions, food merits, etc.” was also considered very important (60%). Personnel and location were considered less important than the other characteristics (40% of owners assigned 1) (Figure 3). 

### 3.3. Customers’ Analysis

Table 2 shows the sample’s sociodemographic characteristics.

Table 3 shows the customers’ preferred hobbies and type of tourism/activities practiced during holidays. Most of the Italian and foreign customers of the selected restaurants declared they prefer cultural tourism (64.4% and 54.9%), followed by seaside tourism (56.3% Italians, 52.9% foreign customers). Moreover, 20% selected the option “visit UNESCO sites”, and 40% “visit cities of art”; like preferred activities during travel, 60% selected “participation to events and expositions”, and 87% “eat local food at typical/traditional restaurants”.

Regarding the sport habits (Table 4) of people interviewed, the results show that both Italian (41.4%) and foreign customers (48.4%) declared they practice a light sport activity, and around 20–25% declared to practice a moderate/strong sport activity. This is an important finding because this sample represents a segment of the population that conducts a good lifestyle concerning non-sedentary behaviors and habits.

Regarding the question “do you know what the MD is” (Table 5), 87.3% of Italian customers and 67.7% of foreign customers declared to know the MD. Unfortunately, when responding to the following question about “what the MD is”, only 36.4% of Italians and 18.0% of foreign customers chose the option “a lifestyle”. Finally, considering the entire sample, only 53% of respondents were aware that the MD is part of humanity’s UNESCO Intangible Heritage. 

**Figure 4:**
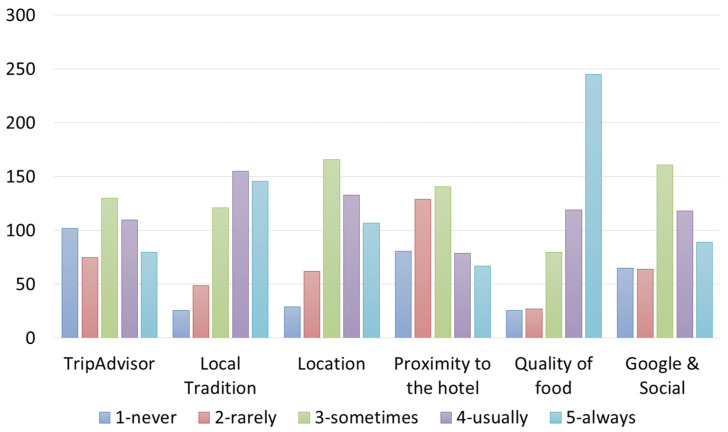
Own processing of the results regarding motivations for choosing a restaurant according to Italian customers interviewed.

**Figure 5:**
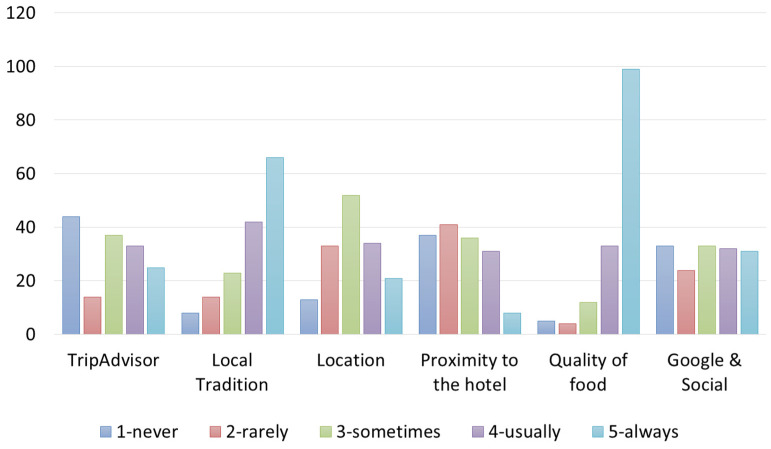
Own processing of the results regarding motivations for choosing a restaurant according to foreign customers interviewed.

Both Italian and foreign customers appeared to pay attention to places where they ate during travels (Figure 4 and Figure 5). Most of the customers declared they “always” choose a restaurant for the quality of food and “usually/always” to eat foods from local tradition. Google search and TripAdvisor were indicated with an almost equal frequency, indicating that there is no tendency to choose or not choose a restaurant based on these types of searches (indifferent), and thus, Google and Trip Advisor are considered only as catalogs, but not as entities, that in a sense accredit a restaurant.

**Figure 6:**
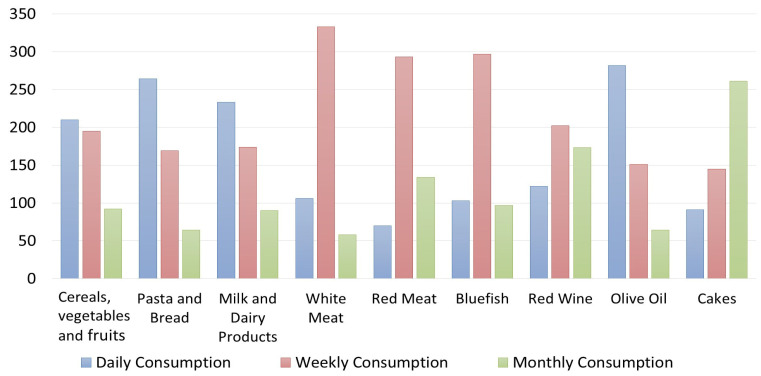
Own elaboration of results regarding the knowledge of the Italian respondents regarding the prescribed frequency of consumption of food groups in the Mediterranean diet pyramid.

**Figure 7:**
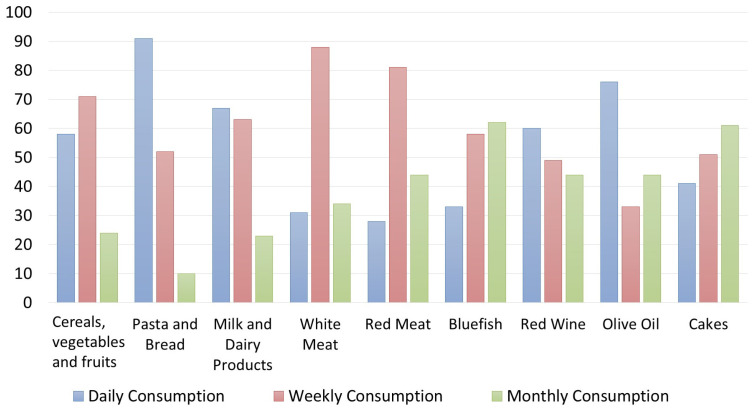
Own elaboration of results regarding the knowledge of the foreign respondents regarding the prescribed frequency of consumption of food groups in the Mediterranean diet pyramid.

Regarding the customers’ knowledge of the MD Pyramid, the results are interesting (Figure 6 and Figure 7). For Italians, the consumption of “pasta and bread” is considered daily, similarly to the consumption of “cereals, vegetables and fruits”. In contrast, the foreign clients interviewed differed in their responses, believing that “cereals, vegetables and fruits” should be consumed mostly weekly, whereas “pasta and bread” should be consumed daily, maybe because they are the two “classical” foods of the Italian cuisine and Italians are the top representatives of the “Mediterranean” cuisine following a cliché of all foreigners. Regarding “red meat”, most of the Italians and foreign clients interviewed are aware that the MD pyramid indicates it must be consumed weekly; only a few respondents said it should be consumed daily or monthly. Red meat, white meat, and bluefish were considered foods to be consumed weekly according to Italians, opposite to foreign customers who do not have correct knowledge about the consumption of bluefish according to the MD pyramid. 

**Figure 8:**
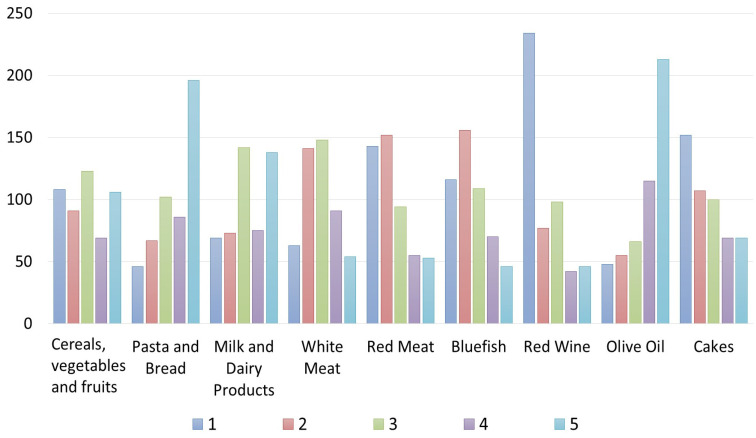
Own elaboration of results regarding the Italian respondents’ consumption frequency of MD foods in the everyday diet. 1—once a week, 2—twice a week, 3—three times a week, 4—four times a week, 5—everyday.

Another interesting result is that, despite the fact that Italians and foreign respondents demonstrated a general correct knowledge of the MD’s food portions, both groups of respondents said they did not adhere to this dietary system in their everyday life. Particularly, Italian customers declared they consume pasta and bread and olive oil every day, white meat from two to four times a week, red meat and cakes one or two times a week, and vegetable and fruit from one to five times a week (Figure 8). This result highlights how the adoption of an eating system or even more so of a lifestyle depends on the subject’s willingness to adhere or not often based on the possession of information, whether correct or incorrect.

**Figure 9:**
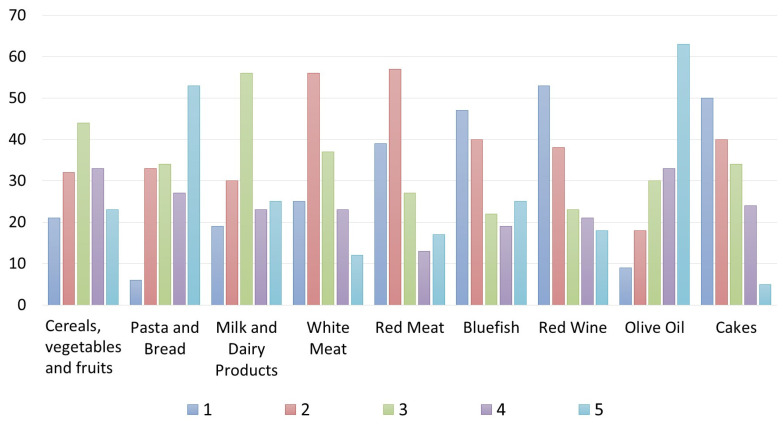
Own elaboration of results regarding the foreign respondents’ consumption frequency of MD foods in the everyday diet. 1—once a week, 2—twice a week, 3—three times a week, 4—four times a week, 5—everyday.

Most foreign respondents claim to eat pasta and bread daily and season the dishes with olive oil. It has been stated by the majority that three times a week, respondents eat cereals and tubers, milk, and derivatives. White meat is consumed by the majority twice a week, along with red meat. According to the analysis, blue fish, sweets, and red wine are consumed only once a week (Figure 9). 

At the end of the questionnaire, customers were asked “if I could live an experience within a Mediterranean Diet Park what kind of activity would you like to attend?”. Most customers responded, “participate in the preparation of dishes”, such as cooking classes, while for all the other activities proposed (i.e., walking, trekking, biking, horse riding, other sports, music events, cultural and art events, eating meals together with other people at the table (typically a classic MD meal)), customers responded in equal measure to all frequencies of very little, little, sometimes, often, and always. This shows that all activities are equally welcome.

## 4. Discussion

According to Ancel Keys’s studies and the UNESCO’s definition, the MD is not solely a healthy dietary pattern, but it concerns a cultural heritage that brings together the over the centuries consolidated lifestyle of people of the Mediterranean basin and their social, traditional and agricultural traditions. Therefore, the MD is a modus vivendi, a way of life that includes the practice of at least light daily physical activity and a way of eating based on the moderation of servings during meals, use of local seasonal products for food preparation, and conviviality. Conviviality is another pillar of the MD as it promotes communal eating and social interaction (slow food). 

To outline and summarize, the MD is:

Physical activity

Moderate and regular physical activity simply means practicing light physical activity (e.g., walking, running, swimming or cycling, gardening, climbing stairs, etc.), for at least 30 min a day and at least 5 days a week. It is very important to prevent many of today’s diseases, such as obesity, hypertension, and atherosclerosis.

Conviviality

Conviviality is the pleasure of being with others. During a convivial meal, people can socialize while eating. Thanks to eating together, the cultural foundation of interpersonal relationships is strengthened, and eating disorders might be prevented.

Seasonality

The seasonality of vegetables is important. In fact, it has been shown that vegetable products in-season have the best commodity and nutritional characteristics. The rhythm of the seasons corresponds better to the body’s needs at that time of year and contributes to the respect for land, biodiversity, and environment. The MD is a sustainable food system that can halt the degradation of ecosystems and preserve plant and animal biodiversity [78].

Local and traditional products

Choosing traditional and local products is a way to protect flavors, recipes, and traditions of a geographic territory from oblivion. It establishes a truly unique relationship of knowledge and taste with traditional cuisine and food production without neglecting the pleasure of food and sometimes discovering the history of our dishes together contributes to learning about the history of Mediterranean communities.

The MD should be enhanced for re-directing the demand of food patterns towards more sustainable ones. However, results of this study revealed a lack of proper knowledge, even among Sicilian restaurants’ clients and owners about what the MD is. Such misinformation is even higher among the Sicilian customers of the sampled restaurants. In fact, the results highlighted that the MD is considered by the majority of the sample solely as a food diet. Particularly, a diet based on the regular consumption of pasta, bread, and other traditional Italian foods without any mention of moderation and restraint of the courses. No information about portions (moderation of meals) and proportions among quantity of calories and food nutrients (carbohydrates, vitamins, minerals, vegetable/animal proteins, saturated/unsaturated fats). Moreover, no information was highlighted about the MD as a sustainable food system [78,79] (e.g., the use of agricultural products according to their natural seasonality). The study conducted in Sicily, highlighted the current situation of misinformation at the various levels of ordinary people and food experts. This result is discouraging after so much talk about the Mediterranean diet at high levels (academics, politicians, cultural representatives) both in Italy and in Sicily. Therefore, it is important to emphasize the relevance of the widespread implementation of communication suitable for different stakeholders of the MD and its importance not only from a health perspective for people, but also for its relevance at the environmental and social levels [78,79,80,81]. In fact, the MD model, despite its undisputed value such that it is considered a World Heritage by the UNESCO, it cannot be imposed on populations; rather, it must be promoted. To do so it appears necessary to convey proper and careful information about the MD with communication at the various levels. This is because, whether the MD model would be known and experienced directly by people, it could be voluntarily adopted and properly disseminated. The promotion of the MD pattern will certainly require different actions and types of informational tools at different levels of depth depending on the target audience [80,81]. Institutional communication by public authorities appears crucial in this case [82], like in other similar cases. 

**Figure 10:**
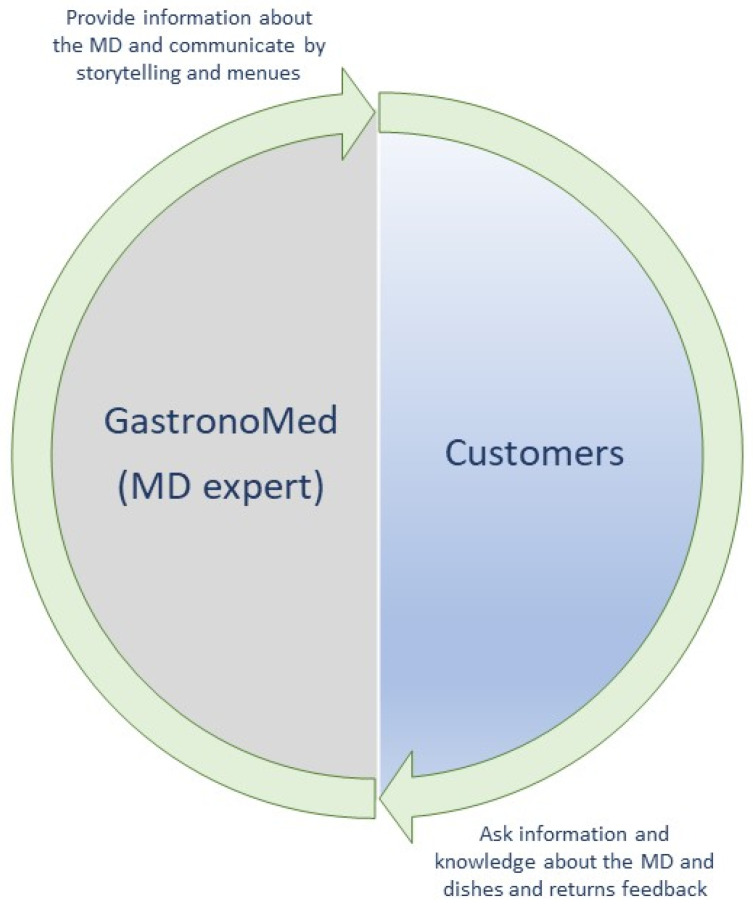
Authors’ conceptualization of the communication flow model between the expert and the restaurant’s clients (input-feedback) about the Mediterranean diet.

Moreover, within restaurants, it might help to train kitchen and dining room staff in storytelling about the MD and the offered dishes that represent it. Through some kind of training, a new professional profile, such as a “Mediterranean Diet expert” could be formed to provide direct information about products (raw materials of foods) to clients and “tell” about the MD in a holistic approach [83,84,85]. Doing so, it will be possible to help cover the gap outcome from the results regarding the chefs/restaurants’ owners lack of knowledge about the agricultural and environmental characteristics of the territory, linked to product seasonality, which make the dishes unique. This expert can be named a “GastronoMeD”. This expert will have to know and communicate production aspects combined with gastronomic processing. The GastronoMeD may use a variety of communication tools based mainly on the existence of a double-way input-feedback model of communication flow between client and expert. This type of communication may adopt two tools to be used as input: storytelling and menus. The communication flow shown in Figure 10 is based on dish storytelling and menus. Particularly, this model (Figure 10) considers the menu an important means for conveying information about the typical dishes of the MD. According to this model, the first step of communication, according to the MD style, starts during the ordering phase. The second step is a discussion with the client during the meal asking feedback on the dishes consumed (conviviality), accompanying customers throughout the entire meal experience. This expert communicates about the MD and the traditional recipes of each specific Mediterranean geographic territory. This will help to satisfy the customers’ interest for local recipes for preparing dishes with foods and doses indicated in the Mediterranean diet model.

### Communication Model with Dimensions by the AGIL Scheme

**Figure 11:**
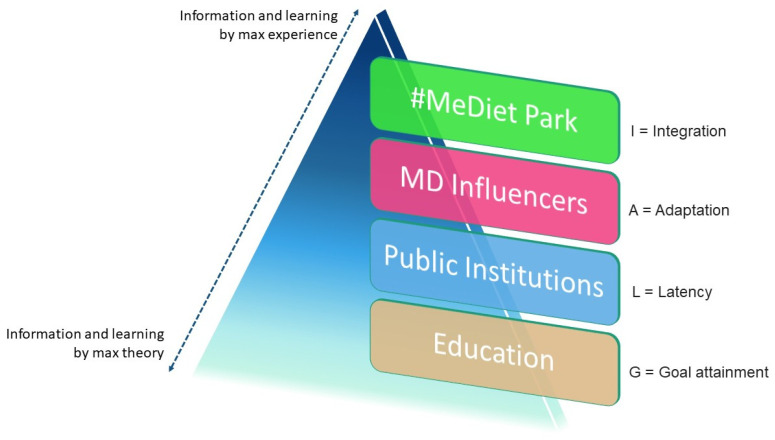
Authors’ conceptualization of the communication scheme about the Mediterranean diet, including the AGIL dimensions.

In this study, an adoptable (open innovation) model of a vertical communication system (VCS) is designed based on the results of the survey. This model can be helpful to policy makers and institutions to convey the knowledge of the MD pattern to each deliverer and user. This model could also provide a useful starting point for policymakers and academics aimed at disseminating information starting from Sicily and Italy (Figure 11). 

The provided model also includes the communication dimensions of the AGIL scheme of Parsons based on both theoretical knowledge and practical experiential knowledge about the MD (Figure 11). 

First, the “Education” of children and young people at schools and specific technical university’s degree courses (like gastronomic Mediterranean sciences) appears relevant [86]. This first step is aimed at providing clear and correct information about the MD pattern and corresponds to the dimension G—Goal attainment of the AGIL Scheme. The implementation of university courses and post graduate master’s degrees will provide expert professionals with specific competences: to be able to properly inform consumers not only at restaurants, but also during seminars or events that concern the MD and food [83,84,85,86]. Among these, the expert on the MD may also be an “ambassador of the Mediterranean diet” and contribute sharing and transferring information and knowledge about the MD to people (like, for example, wine influencers, oenologist, or sommeliers in the wine sector). This expert will be able to recognize the different Mediterranean agricultural products (from vegetable to animal productions, along with their history and use in the preparation of dishes) and communicate the MD model holistically to people all over the world. The GastronoMeD should be an effective communicator/divulgator of the MD not only inside restaurants, but also through social networks and other physical social events where there is a physical participation of people. Storytelling and information about the Mediterranean dishes can add value to the food to be consumed [87,88]. 

The second level of communication of the suggested model involves public institutions. National and regional institutions should cooperate for the establishment of specific information campaigns on the MD, either through schools and universities or also through food events. In fact, as in other similar cases [89,90], the organization of multi-disciplinary events aimed at schoolchildren or ordinary citizens to tell and bring to life what the Mediterranean diet really is would be optimal (seminars, cooking classes, food tastings, and carrying out light physical activities) [89,90]. This type of communication can be expressed with the AGIL dimension of L—Latent pattern maintenance because it tends to create a community around a goal or theme of common interest.

Information and education at schools and universities could also go through food policies that involve the adoption of the MD, also with reference to food served at school and university refectories, cafeterias, and restaurants, as part of integrated projects at the municipal and regional levels, both in smaller territories and at the national level, having specific characteristics for the promotion of food systems that favor short supply chains and the use of local seasonal products [83,84,85,89,90]. These actions could contribute to the improvement of the population’s nutrition and health and, indirectly, to the improvement of environmental sustainability and biodiversity in the Mediterranean area. In this case, Food Policies coordinated between metropolitan cities networked at the national level for the spread of the MD appear very relevant, as it is considered a sustainable model to be applied locally in the various territories of the country [89]. This process should include the involvement of policymakers, academics, teachers, and stakeholders, including food service professionals. In addition, within the framework of local food policies, political institutions should work in collaboration with universities, educational institutions, local agribusinesses, producer associations, and restaurateurs for the implementation of effective integrated actions [89]. 

Recent studies demonstrated that when food is consumed in a pleasant and emotionally satisfying contexts—such as restaurants, hotels, or particularly evocative places (boats, wineries, natural parks, etc.)—perceived satisfaction is higher [56,90]. Social media affects people’ emotions, experiences, and, overall, their behavior. Moreover, nowadays, social networks are used to share pleasant experiences of everyone’s everyday life and also food experiences [56,90]. Lots of literature describes the power of social networks in most economic sectors, such as tourism, fashion, wine, and food [90]. Therefore, social networks also appear to be an effective tool for the communication of the MD model. 

The results of this study showed that the actual information through social networks and websites is mainly about the gastronomic and dietary sphere of the MD model. Nevertheless, at the same time, social networks are a powerful tool of communication used by everyone (young, old, tourists, etc.). Therefore, through social networks, it would be possible to communicate the MD by merging information, knowledge, and experience from institutions and professionals in the field, communicating interactively and instantly sharing content—texts, photos, and videos of moments of conviviality, food, culture, and knowledge of the area—with users. In recent years, storytelling and story-making have become important content creative strategies in communication, in a one-to-many traditional approach (e.g., advertising campaigns). Therefore, the persuasive dimension of communication provided by the AGIL scheme (A—Adaption) appears important at this third step.

Finally, moving gradually from elements of maximum theoretical information to others increasingly characterized by experience, the ultimate expression of communication of the MD imbued with experience could be the implementation of a theme park (I—Integration dimension), possibly following the idea of the existing project in Sicily of the World Park of the Mediterranean Lifestyle. An experiential park, such as a “Mediterranean Diet Park” (MeDiet Park) could be realized [66,91], to enable people to have a real experience of the significance of the Mediterranean diet lifestyle model. This park could also be an attraction for tourists visiting Italy and, at the same time, a powerful means of communicating the MD as a healthy lifestyle based on a sustainable food system, especially for Mediterranean countries [65,66,91]. Within this park, there will be offered the opportunity to carry out different physical, cultural, and gastronomic activities in order to make people understand the MD food-associated habits by living and experiencing them through the practice of behaviors, including diet, rest, sport, conviviality, etc. [91]. With this park, the concept of the MD could be directly linked by visitors with the real place, people, and products of a cultural social group. It will be easy for visitors to qualify and recognize products, places, and people and, in the future, remember the experiences and emotions. In addition, about the food, the “Country of Origin Effect” (COE) and “Made-in” effect will influence visitors (and restaurants’ customers) all over the world [92,93,94]. Therefore, for these reasons, the MD “typical-traditional” food and lifestyle will become an opportunity to enhance healthier behaviors and habits among people. The best practices of local experiences, living labs (generating innovation/research and cooperation), and a community practicing a social approach fostering knowledge on sustainable food systems appears crucial to apply the MD model [93,94].

## 5. Conclusions

The study sought to fill the gap in the literature by providing insight into the current level of information about the MD model among people, both experts in the field and ordinary consumers. The Mediterranean model is a model able to ensure well-being and health according to established principles recognized by precise scientific evidence, which have found an effective representation in the pyramid of the MD. Therefore, understanding this dietary pattern implies knowledge of the geographical, historical, economic, and cultural features of the territorial area to which it refers. The study highlighted critical issues due to a lack of proper information and knowledge about the MD among people. The findings highlighted the need to foster a change in the current perception of the MD as a resource of sustainable development in the Mediterranean at a country and regional level. Moreover, to consolidate the initiative of the World Mediterranean Diet Conference as a permanent forum for multi-stakeholder and transdisciplinary Mediterranean countries, more dialogues and actions appear crucial to accelerate the application of shared healthier food policies in the Mediterranean in a context of social collaboration and peace. 

The role of institutions and academics in delivering correct information about the MD with different actions and communication channels appeared relevant at various levels. The results lead to a reaffirmation of the important notion, already highlighted in a distinguished recent article, that the transformation of food systems requires an integrated collaboration between science, policy, and society [95,96]. Cohesion and cooperation at the national and international level appears important to face the food issues of Mediterranean cities and rural areas [97]. Therefore, the Mediterranean countries’ policy makers should put these themes at the first points of their food policy agenda. This paper, based on the results, provides the first open model of a vertical communication system (VCS) that is adoptable to improve the communication of the MD among people in Mediterranean countries and their knowledge. Moreover, the need of specialized experts (a GastronoMeD) of the MD was highlighted in order to better convey correct and complete information to people about the MD model. 

The limitations are due to the fact this study was carried out in only a region of Italy, although it is the most representative of the MD. Therefore, it would be interesting to repeat the survey in other regions as part of projects, including cross-border projects, to learn about the current level of information and knowledge in other Mediterranean countries to facilitate the promotion of the MD and its adoption by populations.

## Figures and Tables

**Table 1:** Restaurants’ owners.

Variable	Description	(%)
Type of cuisine offered	Local traditional	80.0
	Local organic	11.7
	Fusion/fish	8.3
Customer’s interest for consumption of local dishes/food	High	61.7
	Good	33.3
	Quite	5.0
Self-reported knowledge of the MD	Basic	71.7
	Expert	18.3
	Heard sometimes	10.0
Offers of meal organized according to the MD	Never	6.7
	Rarely	2.3
	Sometimes	22.0
	Often	45.0
	Always	23.0
Request by customers of menus based on MD	Never	28.3
	Rarely	15.0
	Sometimes	40.0
	Often	13.4
	Always	0.3
Is the MD one of the UNESCO Intangible Cultural Heritage of Humanity	Yes	42.0
	No	58.0
Consider the MD a local intangible resource to enhance the Sicilian territory or the Italian food products	Yes, Sicilian Territory	38.3
	Yes, Italian food products worldwide	48.3
	No	-
	Do not know	-

**Table 2:** Sociodemographic composition of the sample.

Variable	Description	Customers’ Country of Origin	
		Italy (%)	Other Countries (%)
Range of age	Under 18	11.2	6.9
	18–30	28.2	25.8
	31–40	14.3	18.3
	41–50	16.7	22.9
	51–60	19.3	12.4
	61–70	7.8	7.8
	Over 70	2.7	5.9
Italian customers’ region of residence *	Sicily	30.0	--
	Other Regions	70.0	--
Foreign customers’ country of origin **	USA	--	20.3
	Australia	--	11.1
	Great Britain	--	11.8
	Germany	--	18.9
	Spain	--	7.8
	Nederland	--	7.2
	France	--	6.5
	Israel	--	3.3
	Other country (Greece, Poland, Sweden, Island, Turkey, Russia)	--	13.1
Education	Secondary school diploma	20.1	29.4
	High school diploma	30.9	18.3
	Bachelor	13.1	18.9
	Master’s degree	26.0	24.3
	PhD	6.4	9.1
	Any other title	3.5	--
Composition of the household	Single	7.9	26.1
	Two adults or elders (no children)	24.7	41.8
	Single/couple with minor children	26.6	19.6
	Single/couple with children over 18	37.8	11.2
	More than two adults or elders (no children)	3.0	1.3
Motivation for traveling	Holidays	69.8	53.6
	Visit relatives and friends	17.1	36.6
	Business	13.1	9.8

* For Italian customers. ** For customers coming from other countries. -- The explanation is that values in this case are not applicable because they are from countries different from Italy.

**Table 3:** Preferred hobbies and type of tourism/activities to carry out during holidays according to restaurants’ customers.

Variable	Description	Customers’ Country of Origin	
		Italy (%) ^1^	Other Countries (%) ^1^
Preferred Hobby	Electronics, informatics	16.3	15.0
	Music, cinema, theater, photo, reading, painting, writing, gardening, etc.	69.6	73.2
	Walking	40.0	52,3
	Competitive sport	21.5	12.4
	Outdoor sports (bike, running, trekking, hiking, etc.)	90.7	72.5
	Indoor sports	23.7	22.9
	Other	3.0	7.8
	No hobbies	2.8	1.3
Preferred type of tourism	Religious tourism	3.0	9.8
	Seaside tourism	56.3	52.9
	Cultural tourism	60.4	54.9
	Sustainable tourism	15.9	23.5
	Sport tourism	20.3	10.5
	Food and wine tourism	22.1	36.6
	Something else	0.8	9.8
Preferred activities during holidays (Italian and foreign respondents together)	Visit UNESCO sites	20	
	Visit cities of art	40	
	Participation to events/expositions	60	
	Go to cinema/theaters/concerts/entertainments	32	
	Visit museums/collections	46	
	Eat local food at typical/traditional restaurants	87	
	Playing sports	33	
	Health and beauty care	26	

^1^ Percentages indicate the preference’s frequency for each of the variables (preferred hobby and preferred type of tourism): the shares do not add up to 100.

**Table 4:** Sport habits of customers.

Variable	Description	Customers’ Country of Origin	
		Italy (%)	Other Countries (%)
Intensity of physical activity practiced	Sedentary	8.5	15.0
	Light	41.4	48.4
	Moderate	25.4	11.1
	Strong	24.7	25.5
Daily time spent for physical activity daily	0–30 min	41.2	54.9
	Up to 1 h	26.6	26.1
	More than 1 h	22.1	11.1
	Competitive sport	10.1	7.8

**Table 5:** Customers’ self-declared knowledge about the Mediterranean Diet.

Variable	Description	Customers’ Country of Origin	
		Italy (%)	Other Countries (%)
Do you know what the Mediterranean Diet is	Yes	87.3	67.7
	No	12.7	32.3
What is mostly the Mediterranean Diet	A healthy diet	40.8	82.0
	A lifestyle	36.4	18.0
Do you think the MD is a humanity’s UNESCO Intangible Heritage (Italian and foreign respondents together)	Yes, it is	53.0	
	No, it is not	40.0	
	I do not know	7.0	

## Data Availability

The data presented in this study are available on request from the corresponding author.
